# Gonorrhœal Arthritis

**Published:** 1910-01-15

**Authors:** 


### Surgery.

### GONORRHŒAL ARTHRITIS.

The fact that an attack of gonorrhœa is not infrequently complicated by inflammatory changes in one or more joints is sufficiently well known, but it is nevertheless easy to overlook the real cause of an arthritis which is really gonorrhœal in origin unless this is constantly borne in mind. The arthritis comes on in a typical case between the third and sixth weeks of the disease, and not necessarily in an attack of gonorrhœa which is acute in itself. Thus there is usually present a gleet, but the patient himself is not aware that there is any connection between the two and makes no mention of the gonorrhœa. In all cases of apparently idiopathic arthritis inquiries should therefore be made as to whether the patient is or has recently been suffering from gonorrhœa. If a discharge is present, it may be taken for granted that the joint is gonorrhœal in origin. Corroborative evidence may often be obtained by a bacteriological examination of the fluid removed from the synovial cavity with an aspirating needle. This is sometimes sterile, but more often than not gonococci may be demonstrated in it; and occasionally in the acuter forms ordinary pyogenic micro-organisms may be found as well.

It is said that gonorrhœal arthritis is commoner in men than in women, but this is probably due to the fact that the presence of gonorrhœa is more manifest in men. At any rate, it will be found in a great number of the cases of apparently unexplained arthritis in women that inter-menstrual vaginal discharge is, or has recently been, present; and it is a curious fact that some form of arthritis is often associated with a leucorrhœa that is not gonorrhœal—that is to say, no gonococci can be found in the discharge microscopically; and further, one occasionally finds cases of synovitis in young women whose menstrual functions are irregular without any discharge at all. This generally yields to a pill containing iron and aloes, or to treatment which restores the regularity of the katamenia.

The affection is usually confined to a single joint, particularly the knee, wrist, and ankle; but hardly any joint is exempt, cases having even been recorded of gonorrhœal inflammation of the temporo-mandibular joint; and not only the joints themselves, but the periarticular structures are also liable to inflammation, particularly the bands of fascia and ligaments. A common example of this is afforded by acute flat foot, which is not really an arthritis, but a gonorrhœal inflammation of the plantar fascia and ligaments of the sole of the foot. This is the explanation of that variety of gonorrhœal arthritis in which the patient complains of stiffness and pain on moving the joint with tenderness on palpation, but no visible abnormality can be discovered on examination. This is the simplest form. The opposite extreme is a very acute suppurative arthritis, clinically indistinguishable from an acute septic arthritis; this is the condition already referred to, in which septic micro-organisms are found in the joint as well as gonococci.

But there are two main forms of a genuine gonorrhœal arthritis—(1) an inflammation which starts as a moderately acute synovitis with swelling of, and effusion into, the joint, which contains turbid fluid. This subsides in whole or in part, leaving a persistent weakness or stiffness due to inflammation of the periarticular structures. (2) The second variety comes on insidiously, without an acute stage, and is characterised by synovial effusion, which persists generally for a long period.

It will be seen that it is the soft structures which are mainly involved, the synovium, the capsule, and the periarticular ligaments and tendons, rather than the ends of the bones which enter into the articulation; and it is open to question whether it would not be better to classify the condition as a "synovitis" or "periarthritis" than as an arthritis proper.

A guarded prognosis should be given, since a gonorrhœal joint is peculiarly resistant to treatment, and convalescence is nearly always prolonged, even if no permanent disability results; while in the acute form, in which suppuration occurs, the very life of the patient may be endangered. It is a curious fact that a patient who has arthritis with one attack of gonorrhœa will almost certainly suffer in the same way if he has the misfortune to incur a subsequent infection, and he should always be warned of this risk.

The primary object in the treatment is to overcome the urethral inflammation, and since this is now chronic it can only be done with injections. It is a much debated question which of the drugs used for urethral medication is the most efficient. In the writer's opinion the most satisfactory results are obtained by the use of a solution of zinc sulphate, gr. ij. to the ounce, or a 2 per cent. solution of protargol. The strength of these may be gradually increased until an appreciable diminution is noticed in the amount of the urethral discharge.

The local treatment must, of course, vary according to the condition of the joint. If a synovial effusion is present, complete rest must be enjoined until this has disappeared. If the effusion is excessive, it may even be necessary to remove it by aspiration. When the fluid has been absorbed or removed, the attention should be concentrated on avoiding permanent weakness or stiffness. Prolonged rest is therefore likely to be harmful as tending to lead to ankylosis. Passive movement and gentle massage should therefore be instituted, a careful watch being kept for evidence of reaccumulation of fluid, in which case these must be desisted from temporarily. In the intervals a counter-irritant is beneficial, and one of the best means of applying this is a Scott's dressing. Its only disadvantage is that it must be taken off and reapplied for each occasion when the limb is massaged. It is hardly necessary to say that in cases in which the joint contains pus the treatment must consist primarily of free drainage of the joint.

